# The Functional Significance of *Mc*MafF_G_K in Molluscs: Implications for Nrf2-Mediated Oxidative Stress Response

**DOI:** 10.3390/ijms242316800

**Published:** 2023-11-27

**Authors:** Ronghui Yao, Longmei Qiu, Li Zhu, Xinglu Chen, Jiaying Zhai, Weifeng Wang, Pengzhi Qi, Zhi Liao, Isabella Buttino, Xiaojun Yan, Baoying Guo

**Affiliations:** 1 National Engineering Research Center of Marine Facilities Aquaculture, Marine Science and Technology College, Zhejiang Ocean University, Zhoushan 316004, China; 17852675309@163.com (R.Y.); qlm2021@163.com (L.Q.); zhuli0318@163.com (L.Z.); chenxx99622@163.com (X.C.); zhaizhai0401@163.com (J.Z.); wangwf@zjou.edu.cn (W.W.); qpz2004@vip.sina.com (P.Q.); liaozhi@zjou.edu.cn (Z.L.); yanxj@zjou.edu.cn (X.Y.); 2 Italian Institute for Environmental Protection and Research (ISPRA), Via del Cedro n.38, 57122 Livorno, Italy; isabella.buttino@isprambiente.it

**Keywords:** nuclear factor erythroid 2-related factor 2, small Maf proteins, *Mytilus coruscus*, protein interaction

## Abstract

The nuclear factor erythroid 2-related factor 2 (Nrf2) is a pivotal regulator of antioxidant gene expression in mammals, forming heterodimer complexes with small Maf proteins through its BZip domain. However, the underlying mechanism of Nrf2 action in molluscs remains poorly understood. The thick shell mussel, *Mytilus coruscus*, represents a model organism for the marine environment and molluscs interaction research. In this study, we used in silico cloning to obtain a small Maf homologue called *McMafF_G_K* from *M. coruscus*. *Mc*MafF_G_K possesses a typical BZip domain, suggesting its affiliation with the traditional small Maf family and its potential involvement in the Nrf2 signaling pathway. Transcriptional analysis revealed that *Mc*MafF_G_K exhibited a robust response to benzo[a]pyrene (Bap) in the digestive glands. However, this response was down-regulated upon interference with *Mc*MafF_G_K-siRNA. Interestingly, the expression levels of Nrf2, NAD(P)H: quinone oxidoreductase (NQO-1), and Glutathione Peroxidase (GPx), which are key players in oxidative stress response, showed a positive correlation with *Mc*MafF_G_K in digested adenocytes of *M. coruscus*. Furthermore, in vitro analysis of antioxidant capacity in digestive gland cells demonstrated that Bap exposure led to an increase in reactive oxygen species (ROS) levels, accompanied by an elevation in total antioxidant capacity (T-AOC), potentially counterbalancing the excessive ROS. Strikingly, transfection of *Mc*MafF_G_K siRNA resulted in a significant rise in ROS level and a down-regulation of T-AOC level. To validate the functional relevance of *Mc*MafF_G_K, a glutathione S-transferase (GST) pull-down assay confirmed its interaction with *Mc*Nrf2, providing compelling evidence of their protein interaction. This study significantly contributes to our understanding of the functional role of *Mc*MafF_G_K in the Nrf2 signaling pathway and sheds light on its potential as a target for further research in oxidative stress response.

## 1. Introduction

In the growth process of aquatic organisms, they are constantly exposed to reactive oxygen species (ROS) and electrophilic substances produced through metabolic and environmental factors [1,2]. These threats can profoundly impact the development and growth of organisms, potentially resulting in tissue damage, senilism, and cell apoptosis. To effectively respond to and counteract these pressures, cells activate multiple defense systems that are closely linked to various cellular processes [3,4,5]. Transcription factors are one of the important contributors to this system, regulating the expression of genes involved in cellular protection [4,6]. One such transcription factor is nuclear factor erythroid 2-related factor 2 (Nrf2), which belongs to the cap’n’collar (CNC) alkaline region leucine zip (BZip) family. Nrf2 plays a significant role in inducing the expression of cell protection genes by directly interacting with antioxidant response elements (ARE) [5,7]. Its downstream target genes include genes encoding antioxidant and detoxification enzymes such as glutathione S-transferase (GST), heme oxygenase-1 (HO-1), quinone oxidoreductase 1 (NQO1), and aldo keto reductase (AKR). These enzymes play a crucial role in the elimination of ROS, xenobiotic metabolism, and detoxification. 

The role of Nrf2 in aquatic organisms has been extensively investigated, shedding light on its significance in maintaining antioxidant defense mechanisms. Research has demonstrated benzo[a]pyrene (Bap) irritation induced the expression of Nrf2 and a series of antioxidant enzymes, such as superoxide dismutase (SOD), glutathione peroxidase (GPX), catalase (CAT), and glutathione reductase (GR), in *Mytilus coruscus* [7]. Similarly, when *Scylla paramamosain* was exposed to stress from *Vibrio parahaemolyticus*, there was a notable increase in the expression levels of *Nrf2* and other antioxidant genes, including *HO-1* and *NQO-1* [8]. In *Litopenaeus vannamei*, the knockdown of *Nrf2* gene using dsRNA-mediated techniques resulted in inhibited antioxidant genes, reduction of antioxidant enzyme activity, upregulated expression of apoptosis and autophagy genes, and noticeable alterations in tissue structure [9]. These findings underscore the crucial role of Nrf2 in orchestrating the response to external stressors in aquatic animals. 

Nrf2 functions in cellular transcription by forming heterodimers with members of the small Maf family, including MafG, MafK, and MafF [10]. Through its BZip domain, Nrf2 establishes these heterodimeric complexes, enabling it to regulate gene expression [11,12]. When cells are exposed to electrophilic agents or ROS, Nrf2 is released from its binding partner, the cytoplasmic protein Keap1, triggering its translocation into the cell nucleus. Once inside the nucleus, Nrf2 forms heterodimers with small Maf, leading to their association with ARE or electrophilic response elements. Remarkable advancements have been made in the study of the small Maf family, particularly with the successful isolation and cloning of *MafG* and *MafK* genes from human hematopoietic cells [12,13]. Subsequent investigations have revealed that MafG and MafK can form heterodimers not only with Nrf2 but also with Nrf1. These heterodimeric complexes play a crucial role in binding to the nuclear erythroid factor 2 (NF-E2) site, further highlighting their regulatory function [14]. Extensive research on the genuine partner molecules involved in supporting *Nrf2* activity in vivo has shown that the absence of small *Maf* can effectively reverse the cellular dysfunction caused by *Keap1* gene deficiency. Furthermore, it has been observed that this absence also leads to a decrease in the mortality rate in mice resulting from *Keap1* loss [10]. These discoveries emphasize the critical collaboration between small Maf and Nrf2 within the organism. Moreover, the absence of *Nrf2* and the lack of small *Maf* both result in highly similar and severe damage in inducing the response of antioxidant and exogenous metabolic enzyme genes to electrophilic agents [15,16]. This further underscores the pivotal role played by Nrf2 and small Maf as heterodimers operating within the intricate biological system. Mice lacking small *Maf* exhibit liver steatosis and gene dysregulation related to lipid and amino acid metabolism, as well as proteasome subunit expression. At the same time, the expression levels of many Nrf2 target genes also decrease [17].

Presently, there have been reports of the successful cloning of small *Maf* genes in aquatic organisms such as *Danio rerio*, *Cristaria plicata* and *Procambarus clarkia*. Takagi et al. [18] identified a novel small Maf protein known as MafT in *Danio rerio*. Further experiments revealed that the co-overexpression of *MafT* and *Nrf2* resulted in synergistic activation of MARE-mediated gene expression in *Danio rerio* embryos [18]. In another study, Wang identified *MafK* in *Cristaria plicata*, confirming the critical detoxification role of *CpMafK* in microcystin toxin stress [19]. In *Procambarus clarkii*, the transcriptional expression of the *PcMafG-like* gene and certain antioxidant genes in the hepatopancreas and gills was significantly up-regulated under Cu^2+^/Cd^2+^ stimulation [20]. However, when *PcMafG-like* was interfered with using dsRNA, the expression of antioxidant genes was inhibited, leading to more severe pathological damage. These findings corroborate the potential of small *Mafs* in eliciting the activation of antioxidant genes in aquatic organisms. Nevertheless, there remains a dearth of research concerning the involvement of small Mafs in the thick shell mussel *M. coruscus*, a crucial model organism in marine environmental studies. *M. coruscus* is primarily distributed in the Yellow Sea and East China Sea, adopting a lifestyle of attachment and filter feeding [21,22]. As they inhabit coastal areas prone to pollution, pollutants accumulate in various tissues, potentially leading to disrupted ROS metabolism [23]. This has profound implications for their survival, growth, development, and evolution, emphasizing the necessity of researching oxidative stress responses in *M. coruscus*. In our previous study, we found that when *M. coruscus* was exposed to acute Bap stress, the Nrf2-dependent antioxidant system was activated at both the transcriptional and enzyme levels [7]. This activation serves as a protective mechanism to counteract the toxic effects of Bap. However, the underlying mechanisms of this potential action, including how small Mafs function, are still unclear. In this study, we focused on investigating the significance of the small *Maf* gene in the Nrf2 pathway and its crucial functional role in the antioxidant process of mussel *M. coruscus*.

## 2. Results

### 2.1. McMafF_G_K Sequence Analysis

**Figure 1:**
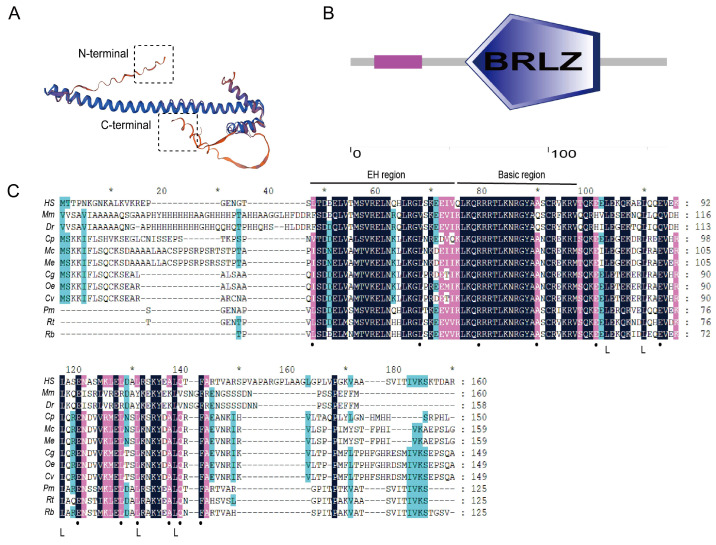
*Mc*MafF_G_K amino acid structure prediction. Note: (**A**): *Mc*MafF_G_K amino acid three-dimensional structure. (**B**): Architecture analysis of conserved domains in *Mc*MafF_G_K using SMART. A conserved BRLZ domain was shown. (**C**): Multiple sequence alignment of *Mc*MafF_G_K. *HS*: *Homo sapiens* MafG sp|O15525.1|; *Mm*: *Mus musculus* Maf NP_001020748.2; *Dr*: *Danio rerio* Maf AAH65941.1; *Cp*: *Cristaria plicata* MafK ATW64756.1; *Mc*: *Mytilus coruscus* MafF_G_K CAC5373282.1; *Me*: *Mytilus edulis* MAFF_G_K CAG2191884.1; *Cg*: *Crassostrea gigas* MafK XP_011432122.1; *Oe*: *Ostrea edulis* MafK-like XP_048760401.2; *Cv*: *Crassostrea virginica* MafK-like XP_022315636.1; *Pm*: *Pecten maximus* MafK-like XP_033732961.1; *Rt*: *Rana temporaria* MafF XP_040215466.1; *Rb*: *Rhinatrema bivittatum* MafF XP_029446021. Identical amino acids are shown in black, while similar amino acids are shown in pink and blue; Circles denote amino acids conserved among small Maf; Heptad repeats of L indicate the leucine zippers.

Complete cDNA sequencing of *Mc*MafF_G_K revealed a composition of 483 nucleotide residues, encoding a 160 amino acid residue protein. Using Expasy, *Mc*MafF_G_K exhibited a molecular weight of 18 kDa and an isoelectric point of 9.85. Structural analysis indicated the presence of an α-helix structure at the C-terminus (Figure 1A). Computational analysis using SMART software (V3.0) infers the existence of a BZip domain within the amino acid sequence (Figure 1B). Furthermore, multiple sequence alignments demonstrated a high degree of conservation in the extended homology (EH) and basic regions, which are crucial for MARE sequence binding (Figure 1C).

### 2.2. Bap Triggered McMafF_G_K Transcriptional Expression

**Figure 2:**
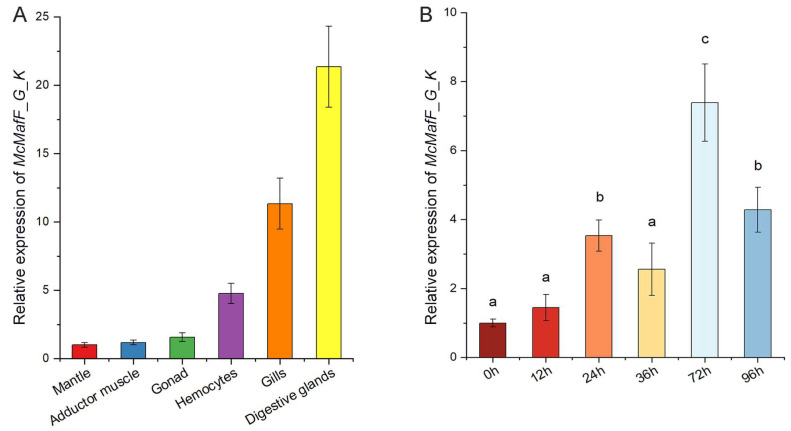
Expression patterns of *MafF_G_K* under Bap stress. Note: (**A**): Expression patterns of *Maf F_G_K* gene in different tissues of *Mytilus coruscus*. Level of expression in Mantle was used as a normalizing factor and set to 1. (**B**): Expression of *MafF_G_K* in digestive glands of *Mytilus coruscus* under Bap stress. The statistical analysis was conducted using a two-way analysis of variance (ANOVA). The vertical bars represent the mean ± SD. (*n* = 3). ^a^ *p* < 0.05, ^b^ *p* < 0.01, and ^c^ *p* < 0.001 compared with the DMSO control group.

The distribution profile of *McMafF_G_K* transcripts in various tissues was investigated using a quantitative real-time PCR (qRT-PCR) assay. Figure 2A demonstrated that the expression level of *McMafF_G_K* mRNA was observed to be highest in the digestive glands, followed by the gills and hemocytes, with the lowest expression detected in the mantle. Furthermore, the transcriptional response of *McMafF_G_K* to exposure to Bap was also examined, as depicted in Figure 2B. Following stimulation with Bap, the transcriptional expression of *McMafF_G_K* exhibited a significant up-regulation starting from 24 h post-stimulus (hps), ultimately peaking at 72 hps. Subsequently, although there was a decrease in *McMafF_G_K* transcription, it remained significantly higher than the control.

### 2.3. McMafF_G_K Showed a Positive Correlation with McNrf2, McNQO-1, and McGPx

**Figure 3:**
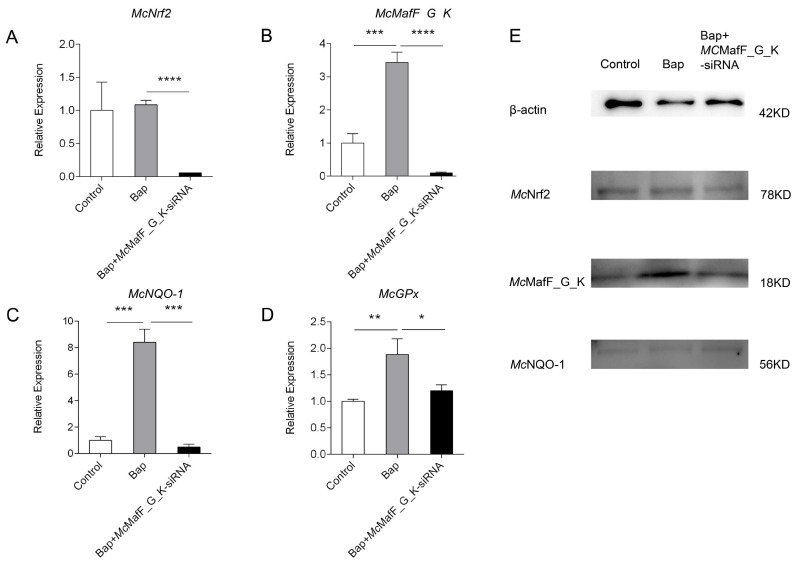
Expression of *Nrf2*, *MafF_G_K*, *NQO-1* and *GPx* genes and proteins in interference experiments. Note: (**A**–**D**): qRT-PCR detection of *Nrf2*, *MafF_G_K*, *NQO-1*, *GPx* mRNA in digested adenocytes after *Mc*MafF_G_K-siRNA interference. (**E**): Detection of Nrf2, MafF_G_K, NQO-1 protein expression in digestive gland cells after *Mc*MafF_G_K-siRNA interference. The statistical analysis employed one-way ANOVA. The vertical bars represent the mean ± SD. (*n* = 3). * *p* < 0.05, ** *p* < 0.01, *** *p* < 0.001 and **** *p* <0.0001 compared with the DMSO control group.

Following a 6 h exposure to Bap, the mRNA expression levels of *McMafF_G_K*, *McNrf2*, *McNQO-1*, and *McGPx* were significantly up-regulated compared to the control group. Subsequently, upon knockout of *McMafF_G_K*, there was a noticeable down-regulation in the expression of *McMafF_G_K* mRNA when compared to the Bap group, suggesting an effective interference effect of *Mc*MafF_G_K-siRNA (Figure 3E). The expression levels of *McNrf2*, *McNQO-1*, and *McGPx* mRNA also showed a significant downward trend once siRNA was added (Figure 3A,C,D). At the protein level, both *Mc*Nrf2, *Mc*NQO-1 and *Mc*MafF_G_K exhibited an up-regulation trend in response to Bap-induced stress, and their expression levels decreased after *Mc*MafF_G_K-siRNA treatment, mirroring the changes observed at the mRNA level (Figure 3E). 

### 2.4. Effects of Antioxidant Capacity

**Figure 4:**
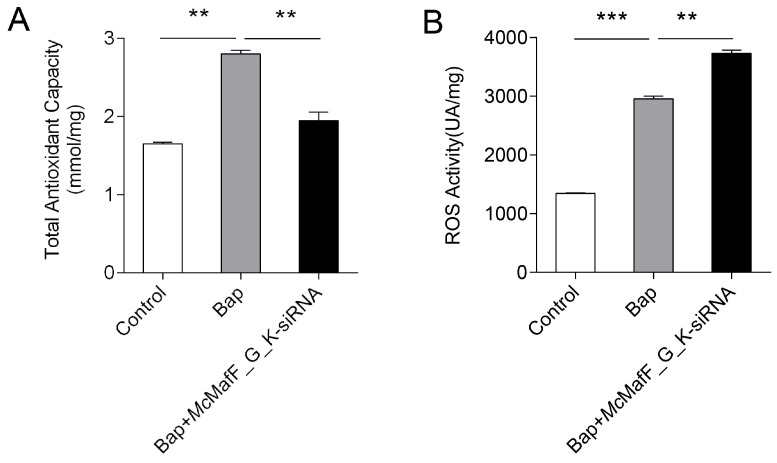
The activity of ROS and T-AOC in digestive gland cells after *Mc*MafF_G_K-siRNA interference. Note: (**A**): The activity of ROS in digestive gland cells after *Mc*MafF_G_K-siRNA interference. (**B**): The activity of T-AOC in digestive gland cells after *Mc*MafF_G_K-siRNA interference. The statistical analysis employed one-way ANOVA. The vertical bars represent the mean ± SD. (*n* = 3). ** *p* < 0.01, and *** *p* < 0.001 compared with the DMSO control group.

Digestive gland cells were isolated and used to investigate the impact of *Mc*MafF_G_K on antioxidant capacity in an in vitro setting. The results revealed that ROS levels significantly increased after 6 h of Bap exposure compared to the control group. Concurrently, the T-AOC level also increased, potentially functioning to counterbalance the excessive ROS (Figure 4). Notably, when *Mc*MafF_G_K-siRNA was transfected into the digestive gland cells, ROS levels exhibited a significant increase (Figure 4B). Furthermore, T-AOC levels displayed a significant down-regulation (Figure 4A).

### 2.5. Protein Interaction between McNrf2 and McMafF_G_K

**Figure 5:**
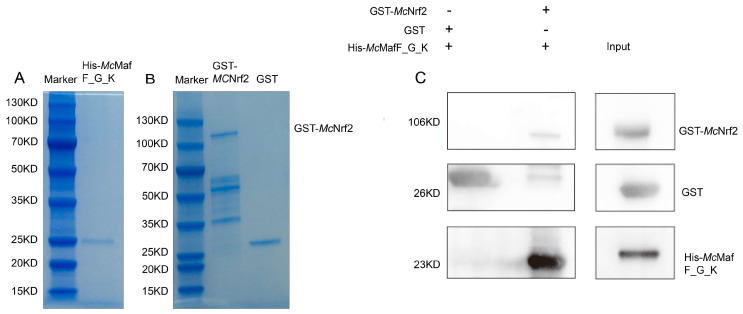
Results of protein purification and GST-Pull down. Note: (**A**): His-*Mc*MafF_G_K protein purification. (**B**): GST, GST-*Mc*Nrf2 protein purification; (**C**): Verification of the interaction between *Mc*Nrf2 and *Mc*MafF_G_K was carried out as follows: In the GST pull-down assay, GST and GST-*Mc*NrF2 were incubated with the His-*Mc*MafF_G_K recombinant protein. The interactions between these proteins were subsequently detected using Western blot analysis with antibodies labelled with His and GST. +: added protein; -: No added protein.

To validate the interaction between *Mc*Nrf2 and *Mc*MafF_G_K, a GST pull-down assay was conducted. The recombinant His-*Mc*MafF_G_K protein was expressed using a prokaryotic expression method and subsequently purified using a Ni-affinity resin (Huiyan Bio, Wuhan, China). The size of the purified His-*Mc*MafF_G_K protein was estimated to be approximately 23 kDa based on the analysis performed on an SDS-PAGE gel (Figure 5A). Similarly, the recombinant GST-*Mc*Nrf2 protein was expressed using the prokaryotic system and purified using a glutathione affinity resin (Huiyan Bio, Wuhan, China). The size of the resulting protein band was observed to be around 106 kDa on the SDS-PAGE gel (Figure 5B). In contrast, the purified GST protein displayed a size of approximately 26 kDa (Figure 5B). Subsequently, the His-*Mc*MafF_G_K protein was incubated with the prey protein, and a Western blot analysis was conducted using anti-GST and anti-His antibodies. The experimental group exhibited distinct bands at 23 kDa (His) and 106 kDa (GST), providing clear evidence of the specific interaction between *Mc*Nrf2 and *Mc*MafF_G_K. In contrast, the control group only displayed a single band at 26 kDa (GST) (Figure 5C).

## 3. Discussion

The Nrf2 pathway, as the key defense system against environmental damage and regulator of body homeostasis, has emerged as a critical research focus. Despite its significance, the understanding of the molecular mechanisms of Maf in aquatic organisms is presently limited. In light of this, our study aims to address this knowledge gap through the identification and characterization of Maf in *M. coruscus*. Our findings revealed that *Mc*MafF_G_K, an important Maf variant, contained a DNA-binding domain and the leucine zipper structures that play a crucial role in both self and other BZip transcription factors’ dimerization [24,25]. Notably, *Mc*MafF_G_K also possesses the HER domain, a universally observed domain in all Mafs. This conserved structural domain facilitates stable DNA binding [26]. These findings substantiate the categorization of the presently identified Maf molecule within the Maf family while illuminating the wider evolutionary backdrop of Mafs. 

The expression profiles of small *Mafs* were comprehensively characterized in various mouse tissues, yielding valuable insights into their differential gene expression patterns. Specifically, *MafK* and *MafF* exhibited prominent upregulation in the lung, underscoring their vital roles in pulmonary physiology, whereas *MafG* displayed pronounced abundance in the heart, highlighting its significance in cardiac function [27]. Furthermore, during hypercapnic stimulation in rats, *MafG* mRNA was discernible not only in the heart but also in diverse tissues, including skeletal muscle, cerebral cortex, cerebellum, liver, stomach, and intestine, suggesting its potential involvement in multiple physiological processes in response to this stimulus [28]. Intriguingly, an exploration of *Maf* gene expression in zebrafish unveiled a widespread distribution of small *Mafs* across different tissues, with the brain exhibiting particularly elevated expression levels, emphasizing the significance of these proteins in neural functions [18]. In *Procambarus clarkia*, the presence of *PcMafG-*like mRNA was detected in all examined tissues, with muscle tissue exhibiting the highest expression levels, likely attributable to the integral role of muscle tissue in the organism’s biology [20]. Additionally, the present study revealed the constitutive expression of *McMafF_G_K* mRNA in all examined tissues, with heightened expression levels observed in digestive glands, gills, and hemocytes, all recognised as immune-associated tissues in bivalves, implying their potential contributions to immune responses. These comprehensive analysis of small *Maf* gene expression profiles in different tissues provides valuable insights into their differential regulation and tissue-specific significance. These findings contribute to our understanding of the multiple physiological roles played by small Mafs. 

Small Mafs play a crucial role as transcription factors in the activation of ARE-dependent genes, which are responsible for cellular protection, effectively shielding the organism from environmental harm, and this is essential for *Nrf2*-mediated gene activation. Research has shown that the expression of ARE-dependent genes in the liver of mice is influenced to varying degrees and exhibits high sensitivity to oxidative stress responses. Inducers such as hydrogen peroxide, ARE or electrophile responsive element (ARE/EpRE) inducers, and hypercapnia (elevated carbon dioxide levels) can stimulate the induction of small Mafs, activating the expression of ARE-dependent cell protective genes [16,28,29]. In a study investigating whether inducers can also activate the expression of zebrafish small Mafs, it was found that diethylmaleate (DEM) can induce the expression of *MafT* and *MafG1*. Additionally, the co-expression of *MafT* and *Nrf2* synergistically activates gene expression mediated by MARE in zebrafish [18]. Upon Cu^2+^/Cd^2+^ stimulation, the expression of *PcMafG-*like and downstream antioxidant genes is upregulated in the hepatopancreas and gills of *Procambarus clarkia* [20]. During this study, we observed an upregulation in the expression levels of *McMafK_G_F*, *McNrf2*, and downstream target genes *McGPx* and *McNQO-1* in the digestive gland cells of *M. coruscus* following a six-hour Bap exposure. At the same time, we used qPCR technology to assess gene expression after *McMafF_G_K* gene interference. The results showed significant downregulation in the expression levels of *McMafK_G_F*, *McNrf2*, *McNQO-1*, and *McGpx* compared to the Bap stimulation group. Specialised research into *Mafs* regulation unveiled that mice embryos with reduced *Maf* expression exhibited a decreased basal expression of ARE-dependent cellular protective genes. This decreased expression of oxidative stress response genes may exacerbate cell apoptosis, embryonic growth retardation, and impaired liver function [30]. To clarify the precise contributions of small Mafs to the functions of various CNC proteins, a mouse strain lacking small Mafs in the liver was established. It was found that the livers of small Maf-deficient mice shared a similar expression profile with those of Nrf1 and Nrf2-deficient mice. This was characterised by a reduction in the expression levels of Nrf2 target genes, along with liver fat degeneration and gene dysregulation related to lipid and amino acid metabolism, as well as proteasome subunits [17]. These findings are consistent with the results of this study, indicating that Mafs, as central regulators of the Nrf2 signaling pathway, are indispensable in the process of regulating the basal expression of ARE-dependent cellular protective genes during oxidative stress.

The continuous generation of ROS is a common response in aquatic animals when facing external stressors. Both non-biological water pollution and biological factors can induce the excessive accumulation of ROS in marine organisms, leading to oxidative stress. In our previous study, we observed that exposure to Bap significantly increased the activities of SOD, CAT, GPx, and GR enzymes in the digestive glands of *M. coruscus*. This suggests that upon exposure to Bap, the antioxidant defense system is triggered to counteract oxidative stress, effectively protecting cells from BaP-induced oxidative damage [7]. Huang et al. [9] found that injecting Nrf2 dsRNA into *Litopenaeus vanname*i resulted in decreased activities of SOD, CAT, and GPx enzymes, while malondialdehyde (MDA) activity increased. Similarly, Wang et al. [31] demonstrated that silencing Nrf2 for 72 h using RNA interference in the digestive gland of *Cristaria plicata* led to significantly higher levels of MDA, indicating elevated lipid peroxidation compared to the control group. In the binding and recognition process of the Nrf2/Maf heterodimer for activating the antioxidant stress response, Nrf2 and Maf play a crucial synergistic role. Interfering with Nrf2 and Maf may lead to similar results [10,32]. Additionally, it was observed that when discussing the significant role of MafG in the *Procambarus clarkii* oxidative response, the activities of GSH, Cu/Zn-superoxide dismutase (CZ-SOD), and CAT significantly increased in the Cu^2+^/Cd^2+^ stimulation group, while interference with *Pc*MafG-like led to a reduction in enzyme activities [20]. In this study, Bap stimulation resulted in a significant increase in T-AOC and ROS activities, indicating that Bap disrupts the redox balance within the organism, leading to the accumulation of ROS. *M. coruscus* alleviates the damage caused by ROS accumulation by enhancing its total antioxidant capacity. However, *Mc*MafF_G_K siRNA interference resulted in increased ROS activity and decreased T-AOC activity, suggesting that Bap activates the Nrf2 signaling pathway to mitigate oxidative stress, and silencing MafF_G_K disrupts the normal antioxidant mechanisms, resulting in decreased total antioxidant capacity and elevated ROS levels. These results suggest that small Mafs may play a crucial regulatory role in the antioxidative stress process of bivalves, and their functional loss could potentially exacerbate damage to the organisms. 

Small Mafs and Nrf2 belong to the CNC transcription factor family, both possessing a BZip structure that mediates DNA binding and dimerization [15,24,25]. The Nrf2-small Maf heterodimer plays a crucial role in oxidative stress responses by transcriptionally activating a multitude of cell-protective genes through antioxidant response elements [33]. Surface plasmon resonance microarray imaging technology has been employed to demonstrate the high affinity of the Nrf2-Maf heterodimer for MARE-like elements [33]. In the context of the regulation of *C. plicata* Prx5 within the Nrf2/ARE signaling pathway, mass spectrometry analysis has identified MafK protein as one of the interactors with Nrf2-BZip, which was validated through yeast two-hybrid experiments [5]. Herein, we present the successful construction of recombinant vectors His-*Mc*MafF_G_K and GST-*Mc*Nrf2, as well as the expression of the recombinant proteins in vitro. Our GST-pull down experiments have confirmed the interaction between *Mc*Nrf2 and *Mc*MafF_G_K, thus highlighting the formation of heterodimers between *Mc*MafF_G_K and *Mc*Nrf2 that participate in the transcriptional regulation of antioxidant stress responses.

## 4. Materials and Methods

### 4.1. Animals

A total of 200 *M. coruscus* individuals were collected from Donghe Market in Zhoushan city, Zhejiang province. They were then temporarily cultured for a week in seawater with a temperature of 25 ± 1 °C and salinity of 30‰. During the domestication process, 50% of the seawater was replaced daily, and spirulina powder was fed to them every day. 

### 4.2. Silicon Cloning for McMafF_G_K

The sequence information of *McMafF_G_K* cDNA was silicon cloned from the National Center for Biotechnology Information (NCBI) (accession number: CAC5373282). A specific primer pair (Table 1) was designed to amplify the sequence of the *Mc*MafF_G_K open reading frame (ORF) region. After DNA sequencing of the PCR products, the putative amino acid sequence was presumed. Theoretical calculations of the isoelectric point and predicted molecular weight of *Mc*MafF_G_K were carried out utilizing the EXPASY platform (V3.0, http://web.expasy.org, accessed on 24 September 2023). To predict the presence of conserved domains, we employed the SMART tool (V3.0, http://smart.embl-heidelberg.de/, accessed on 15 October 2023). Homologous amino acid sequences of *Mc*MafF_G_K were sourced from NCBI utilizing their Blast service (https://www.ncbi.nlm.nih.gov/blast, accessed on 13 October 2023).

### 4.3. Bap Exposure

The Bap exposure assay was conducted following the methodology described in our previous publication [7]. In brief, 15 mussels assigned to the experimental cohorts were subjected to a 50 μg/L concentration of Bap, while 3 in the control cohort were solely exposed to the vehicle DMSO at an equivalent dosage. Subsequent to stimulation, digestive glands were dissected at multiple time intervals (0, 12, 24, 36, 72, and 96 h). To minimize the impact of individual variability, digestive gland samples from three mussels were pooled together as a single replicate, and three replicates were conducted for each time point. Additionally, six different tissues, including the adductor muscle, gills, mantle, gonad, hemocytes, and digestive glands, were extracted from nine untreated, healthy mussels to investigate the tissue distribution of *McMafF_G_K.*

### 4.4. Cell Transfection

Digestive gland tissue weighing 0.3–0.35 g was carefully disinfected by immersing it in 25 mL of Hank’s solution (Solarbio, Shanghai, China) supplemented with 1% penicillin-streptomycin-gentamicin solution (Solarbio, Shanghai, China). Thereafter, the tissue was thoroughly rinsed with PBS and finely minced using surgical scissors. To facilitate digestion, 8 mL of 0.25% trypsin solution (Solarbio, Shanghai, China) was added and allowed to react for 20 min with vigorous shaking and stirring every 5 min. After the hydrolysis was terminated, the resulting filtrate was collected and centrifuged at 3000 rpm for 15 min at 4 °C. Subsequently, the cell precipitate was suspended in L-15 medium (Solarbio, Shanghai, China) containing 10% fetal bovine serum (Solarbio, Shanghai, China). The resultant solution was evenly distributed onto a six-well plate and cultured in a cell incubator at 28 °C. Transfection with *Mc*MafF_G_K-siRNA using GP-Transfect-Mate (Genepharma, Shanghai, China) was performed when the cell density reached 60% to 80%. To evaluate the impact of *Mc*MafF_G_K on the Nrf2 pathway, the cells were then exposed to Bap (50 μmol/mL) for a period of 6 h.

### 4.5. qRT-PCR

qRT-PCR assays were performed conventionally. Total RNA was extracted from the tissues and cells using Trizol (Invitrogen, Carlsbad, CA, USA) following established protocols. The extracted RNA was subsequently converted into cDNA using the Go Script^TM^ Reverse Transcription System kit (Promega, Madison, WI, USA). For gene detection, the SYBR Green Master Mix, included in the TB Green^®^Fast qPCR Mix Kit (TaKaRa, Kusatsu, Japan), was employed on the 7500 system. The experiment utilized specific primers, as detailed in Table 1. Each sample was analyzed in triplicate, and gene transcription levels were quantified using the 2^−ΔΔCt^ method. Finally, data were statistically analyzed and visualized using SPSS 19.0 and GraphPad Prism 8.0.

### 4.6. Western Blotting

The Western blot analysis followed the protocol outlined in our previous study [34]. Initially, cultured cells were lysed using RIPA buffer (Beyotime, Shanghai, China), and the protein concentration was quantified using a BCA protein assay kit (Beyotime, Shanghai, China). Subsequently, 20 μg of total protein was isolated and separated on 8% and 12% SDS-PAGE gels and then transferred to a PVDF membrane. After blocking with 5% skim milk at room temperature for 1 h, the membrane was incubated overnight at 4 °C with primary antibodies (HuaBio, Hangzhou, China, 1:500). Following three washes, the membrane was incubated with a horseradish peroxidase-conjugated secondary antibody (ABclonal, Wuhan, China, 1:500). The target protein bands were visualized using the enhanced chemiluminescence (ECL) system (Beyotime, Shanghai, China) and subsequently normalized to β-actin.

### 4.7. McMafF_G_K Recombinant

The prokaryotic expression system pet28-*Mc*MafF_G_K was successfully transformed into *Escherichia coli* BL21 host cells (Qingke, Beijing, China) to initiate protein expression. To induce the expression of His-*Mc*MafF_G_K protein, the transformed cells were incubated at 37 °C for 4 h with 0.2 mM isopropyl β-D-1-thiogalactopyranoside (IPTG) (Sangon Biotech, Shanghai, China). Similarly, the plasmid pGEX-4T-1-*Mc*Nrf2 was transformed into TSsetta (DE3) (Qingke, Beijing, China) and allowed to culture overnight at 25 °C. The induction of GST-*Mc*Nrf2 recombinant protein expression was carried out using 0.2 mM IPTG (Sangon Biotech, Shanghai, China). Following cell culture, the culture medium was centrifuged to remove the supernatant. The bacterial cells in suspension were then subjected to sonication for disruption, followed by centrifugation to separate the supernatant from the pellet. Subsequently, the protein samples were verified through 8% and 12% SDS-polyacrylamide gel electrophoresis. The purification of His-*Mc*MafF_G_K and GST-*Mc*Nrf2 proteins was achieved by utilizing Ni-affinity resin and glutathione affinity resin (Huiyan Bio, Wuhan, China), respectively. Solution exchange was performed using Amicon^@^Ultra-15 ultrafiltration tubes (Millipore, Rockland, MA, USA). Finally, to prevent protein degradation, the His-*Mc*MafF_G_K and GST-*Mc*Nrf2 proteins were stored in Bup^TM^ Tris-buffered saline (TBS) (Thermo Fisher Scientific, San Jose, CA, USA) supplemented with 1 mM phenylmethylsulfonyl fluoride (PMSF) (Sangon Biotech, Shanghai, China).

### 4.8. GST Pull Down

In the GST-pull down experiment, 100 μg of GST-*Mc*Nrf2 protein was initially combined with glutathione agarose resin and gently oscillated at 4 °C for 1 h. Following this, 150 μg of His-*Mc*MafF_G_K protein was added to the GST-*Mc*Nrf2 solution, and the mixture was gently oscillated at 4 °C for 2 h. The eluate was collected and then heated at 95 °C for 5 min for subsequent Western blotting analysis. For Western blotting, protein separation was performed using a 4–20% linear gradient SDS-polyacrylamide gel (GenScript ProBio, Billerica, MA, USA). After electrophoresis, the proteins were transferred onto a membrane using a standard transfer protocol. The membrane was then subjected to blocking using skim milk powder to minimize non-specific binding. The membrane was then incubated overnight at 4 °C with mouse monoclonal anti-GST (1:10,000) and anti-His (1:10,000) antibodies (Sanying Bio, Wuhan, China). The membrane was washed three times with TBST to remove any unbound antibodies. Following that, the membrane was exposed to a secondary antibody conjugated with horseradish peroxidase (Sanying Bio, Wuhan, China, 1:30,000) at room temperature for 1 h. The bands corresponding to the target proteins were visualized using the ECL system.

### 4.9. Enzyme Biometry

Enzyme activity tests are based on our previous research [35]. The measurement of ROS levels was performed according to the instructions provided with the ROS detection kit (Jiancheng, Nanjing, China). In brief, cells were collected and suspended in PBS. After incubation with the 7-Dichlorofluorescin Diacetate probe (10 μmol/L) at 37 °C for 1 h, the cells were collected and resuspended in PBS. Subsequently, the fluorescence intensity was determined using a fluorescence spectrophotometer. The protein content in the cell samples was measured using the BCA protein concentration determination kit (Beyotime, Shanghai, China), and the final results were expressed as fluorescence units per milligram of protein.

The assessment of T-AOC was conducted using the Total Antioxidant Capacity Detection Kit (Beyotime, Shanghai, China) based on the 2,2′-azino-bis (3-ethylbenzothiazoline-6-sulfonic acid) (ABTS) method. Following the collection and washing of cells with PBS for the subsequent detection step, the supernatant was transferred to a 96-well plate, and the ABTS working solution was added. Subsequently, the 96-well plate was incubated at room temperature for 6 min and measured using the Spark^®^ Multimode Microplate Reader (V2.3). The protein content in the cell samples was determined using the BCA protein concentration determination kit (Beyotime, Shanghai, China China). The total antioxidant capacity of the samples was determined by referencing the standard curve of the Trolox standard solution provided in the kit. The final results of total antioxidant capacity are typically expressed as mmol/mg or mmol/g of protein weight.

### 4.10. Statistical Analysis 

The mean values and standard deviation (SD) were used to describe the data. Statistical significance was set at a *p-*value less than 0.05 (*p* < 0.05). Group differences were assessed using either one-way ANOVA or two-way ANOVA. Statistical analyses were conducted using GraphPad Prism software version 6.0.

## 5. Conclusions

*Mc*MafF_G_K, a homologue of small Mafs, was computationally identified in *Mytilus coruscus*. Transcriptional analysis revealed the significant response of *Mc*MafF_G_K in the digestive glands to Bap, which was down-regulated upon interference with *Mc*MafF_G_K siRNA. Notably, a positive correlation was observed between *Mc*MafF_G_K and key players involved in oxidative stress response. Intriguingly, transfection of *Mc*MafF_G_K-siRNA resulted in a substantial increase in ROS level and a down-regulation of T-AOC. The functional relevance of *Mc*MafF_G_K was further validated through a GST pull-down assay, confirming its interaction with McNrf2 and providing compelling evidence of their protein interaction. Overall, this study provides valuable insights into the functional role of *Mc*MafF_G_K in the Nrf2 signaling pathway and its implications for oxidative stress response in molluscs.

## Figures and Tables

**Table 1:** PCR primer pairs used in the present study.

Primer	Sequence	Usage
*β-actin*	AACGCTTCACGAATTTGCGT	Internal reference for qPCR
	ATGAAACCACCTACAACAGT	
*McNrf2*	ATGCCAACCCATGTAGAGCC	For *McNrf2* qPCR
	TACCATCGTTCCAAACT	
*McGPx*	TCAACGGAGAAAAGGAACAT	For *McGPx* qPCR
	TGTTTGTGATCCCTCCTCTA	
*McNQO-*1	CCGATGACAAACAGAGAGAA	For *McNQO-1* qPCR
	CGTTCATGTCTCCACATACT	
*McMafF_G_K*	AGGTGAAAAAGATGGGTTGA ATGTGGGAAACCAGTTGAAT	For *McMafF_G_K* qPCR
SiRNA-*Mc*MafF_G_K	GUUGCUUAAAGGGUUAAAUTT	For *McMafF_G_K* gene silencing
	CCACCCAUUAUGUAUUCAATT	

## Data Availability

Requests for access to the data, statistical code, questionnaires, and technical processes may be made by contacting the corresponding author at guobaobao2000@126.com; guobaoying@zjou.edu.cn.
